# Evaluation of anhydrous processing and storage methods of the temperate bacteriophage ɸV10 for integration into foodborne pathogen detection methodologies

**DOI:** 10.1371/journal.pone.0249473

**Published:** 2021-04-06

**Authors:** Andrew Kanach, Theresa Bottorff, Min Zhao, Jun Wang, George T. C. Chiu, Bruce Applegate

**Affiliations:** 1 Department of Biological Sciences, Purdue University, West Lafayette, Indiana; 2 Purdue University Interdisciplinary Life Science Program (PULSe), West Lafayette, Indiana; 3 School of Electrical and Computer Engineering, Purdue University, West Lafayette, Indiana; 4 Department of Food Science, Purdue University, West Lafayette, Indiana; 5 College of Food Science and Technology, Qingdao Agricultural University, Qingdao, China; 6 School of Mechanical Engineering, Purdue University, West Lafayette, Indiana; Texas A&M University College Station, UNITED STATES

## Abstract

Due to the nascency of bacteriophage-based pathogen detection technologies, several practical hurdles stand in the way between providing promising proof-of-concept data and development of robust detection platforms. One such hurdle, and the focus of this work, is the development of methods for transitioning laboratory stocks of bacteriophage into functional, consistent, and shelf-stable delivery methods in commercial detection kits. Research described here was undertaken to evaluate two methods for their ability to store the bacteriophage ɸV10 at ambient temperature without aqueous storage solutions while limiting loss of viability. ɸV10 is a temperate bacteriophage which solely infects the zero-tolerance food adulterant *Escherichia coli* O157:H7 and has been genetically modified to generate a detectable phenotype in host cells. In order to integrate this reporter bacteriophage into food-borne pathogen detection methodologies, two methods of processing phage suspensions for long-term, ambient storage were evaluated: printing solutions onto pieces of dissolvable paper and lyophilizing suspensions with sucrose. Applying phage to dissolvable paper yielded key attributes to consider when addressing phage viability, however, optimized methodology still resulted in an approximate five-log reduction in titer of viable phage. Lyophilization of ɸV10 with various concentrations of the cryoprotectant molecule, sucrose, yielded losses of approximately 0.3-log after 120 days of storage at 23°C. Liquid storage buffer samples with and without sucrose saw a reduction of viable phage of at least 3.9-log in the same period. Additionally, the ability for ɸV10 to form lysogens in an *E*. *coli* O157:H7 host was not negatively affected by lyophilization. Drying ɸV10 at ambient temperature drastically reduces the viability of the phage. However, lyophilizing ɸV10 in the presence of sucrose is an effective method for dehydration and storage of the phage in ambient environmental conditions for an extended time lending to commercial application and integration into foodborne pathogen detection methodologies.

## Introduction

Foodborne illnesses are an extremely deleterious problem that the food industry is obligated to mitigate. One common food-borne pathogen is *Escherichia coli* O157:H7, which has been the cause of many notable outbreaks such as the romaine lettuce outbreak of 2019 [1]. The severity of illness and economic repercussions associated with this food-borne pathogen led the USDA to classify it as an adulterant, meaning the presence of a single detectable *E*. *coli* O157:H7 cell within a food sample calls for the entire lot of food product to be destroyed [2]. With these costs to the health and food industries, the development of rapid and inexpensive detection methods is a prominent focus in these sectors.

Bacteriophage (or “phage”) are viruses that specifically infect bacteria and inherently utilize one of two strategies for replication: a virulent lifecycle or a temperate lifecycle [3]. Virulent (lytic) phages reproduce by inserting their genome into a bacterial cell, replicating their genomes and proteins to form mature virions, and lysing the host to release progeny phage [4]. Temperate (lysogenic) phage can replicate their genome in two ways: by undergoing lytic cycle as virulent phage do, or by integrating their genome into the genome of the bacterial host, thus becoming a prophage [4]. While in this state, the phage genome is replicated with the host chromosome and is included in any resulting daughter cells of the host. This stage of infection is where recombinant reporter systems may be utilized to generate detectable signals which are enhanced as host cells multiply in an enrichment setting. Temperate prophage may also spontaneously switch to the lytic lifestyle and kill the host cell while releasing dozens to thousands of progeny phage [5, 6].

Currently, the vast majority of phage-based products which are available commercially are designed around a mixture of several lytic phage with the goal of controlling bacterial populations, rather than detecting them. Within the United States, liquid lytic phage suspensions have been FDA-approved for use in the food industry to control *E*. *coli* and *Salmonella* spp. [7, 8]. Additionally, recombinant phage technologies have been developed and patented for detecting the common foodborne pathogen *Listeria* spp., as well as *E*. *coli* O157:H7 [9, 10]. As phage-based technologies emerge, reliable strategies for packaging and integrating the phage technology into pathogen detection methodologies will be crucial for their adoption by the industry. Reliability and stability of the phage components of a detection method are critical features for independent approval of phage-based technologies, which to this point, has been very rare [11]. Low rates of natural lysogeny and limited signal production limit new temperate phage-based detection technologies, but emerging research suggests these technologies are imminent [12–14].

The development of temperate reporter phage for the detection of target bacteria (e.g. food-borne pathogens) provides some unique advantages over other detection methods and can leverage the requirement of an initial enrichment step to increase the number of target bacteria within a sample. As with lytic phage, temperate phage benefit from strain-specific targeting of only living bacteria, but because temperate phage integrate their genomes into hosts without killing them, targeted bacteria may also be isolated and further characterized after detection. ɸV10 is a temperate coliphage in the family *Podoviridae* and specifically infects *E*. *coli* O157:H7 using the O-antigen as its target site [15]. A recombinant reporter phage, ɸV10*nLuc*, was developed for the targeted infection, integration, and detection of the food-borne pathogen *E*. *coli* O157:H7 [16]. The gene for the optimized luciferase enzyme nanoLuc® (Promega Corporation) was homologously recombined into the wildtype ɸV10 genome, resulting in a substantially increased rate of lysogeny (59.6%) and infected cells that are bioluminescent upon the addition of a furimazine (2-furanylmethyl-deoxy-coelenterazine) substrate [13]. As more phage like ɸV10*nLuc* are in development, the step of adapting them for commercial bacteria detection platforms becomes the next challenge to address.

Several phage-based pathogen control systems are commercially available, but the development of temperate phage-based pathogen detection systems necessitates evaluation for how to integrate them into the detection workflows. Many lytic phage products are highly concentrated liquid suspensions, which require refrigeration to enhance the product shelf-life. In the settings in which these products are applied, liquid formulations of phage are suitable as the products are frequently applied to surfaces or added to food of livestock animals to infect and kill target bacteria. However, the use of temperate phage for purposes of detecting target bacteria requires consistency in the number of phage used in a detection assay; too few phage in a detection assay may result in delayed signal production, too many phage in a detection assay may result in a false negative outcome due to spontaneous lytic infection of a very low presence of the target bacteria (e.g. one or two cells in a sample). In addition, temperate phage would likely be added to an enrichment culture for an environmental sample if being used to detect target bacteria. To minimize the labor required to add temperate phage and to limit the risk of sample contamination, a “hands-off” method for addition of phage would be a marketable attribute. One approach to functionalizing phage has been to use precisely controlled printing technologies to apply phage to paper materials [17–19]. However, the longevity of viable temperate phage from these treatments has not been assessed, thus, there is a need for reliable methods to functionalize and stably store phage for bacterial-detection purposes.

Lyophilization (or freeze drying) has historically been a valuable technique to microbiology as it has been a reliable method for preserving samples and strains [20, 21]. This method has also been applied to maintaining a variety of bacteriophages in lab strain collections over several years [22]. Many studies focused on the viability of phage after freeze drying have concluded that in some cases, viability can be maintained for years with minimal loss of titer, while in other cases, viability of phage stocks can be entirely lost [23, 24]. Merabishvili, *et al*. (2013) concluded that several additives to liquid phage stocks can generate improved phage viability compared to stocks of phage in only storage buffer. One of the most effective additives was sucrose (0.1M and 0.5M), which showed a loss in titer of approximately 2 logs over the course of 37 months at 4°C, an acceptable loss for phage stocks which contain concentrations of phage at 10^8^–10^11^ pfu*mL^-1^. Ultimately, the lack of knowledge on storage processing of ɸV10 and ambient temperature storage methods of phage in general represent gaps in the larger literature body and were primary motivations for this work.

Bacteriophage represent one potential future for the field of foodborne pathogen detection; their host specificity, live versus dead target cell distinction, low cost of production, and ability to customize detectable signals through genetic engineering represent tangible improvements over current methodologies. However, any future successful integration into mainstream testing protocols is dependent upon reliable and stable methods of processing, storing, and physically adding the reporter phage. Current phage-based technologies focus on controlling (or eliminating) bacterial populations, rather than detecting them, and are packaged in liquid suspensions that require refrigeration. Thus, the overall aim of this research was to identify suitable methods for storing, packaging, and delivering a temperate phage (ɸV10) for application without need for refrigeration or water in order to facilitate bacteriophage integration into foodborne pathogen detection protocols.

## Materials and methods

### Media preparation and strains

Luria Bertani (LB) broth (pH 7.5) was prepared by adding 10g tryptone (Fisher Scientific, Pittsburgh, PA), 5g yeast extract (Fisher Scientific), and 10g NaCl (Fisher Scientific) to 1L deionized (DI) water, then autoclaving. LB plates were made by adding 17g of agar (Fisher Scientific) to LB broth before autoclaving. For plates containing kanamycin, 1mL of a filter-sterilized stock of kanamycin sulfate was added to LB Agar after autoclaving and tempering to 45°C to a final concentration of 50ug per mL. LB top agar was made by adding 6g*L^-1^ agar to LB broth and steamed until the agar dissolved. Modified LB top agar was made as LB top agar above but included 0.02% w/v Coomassie Brilliant Blue G250 and 5% v/v glycerol (Alfa Aesar, Ward Hill, MA). After steaming, 4mL aliquots of molten top agar were pipetted into 16mm x 100mm borosilicate glass tubes (Fisher Scientific), capped, and autoclaved.

Phosphate buffered saline (PBS) was made by dissolving 1.2g Na_2_HPO_4_ (Sigma Aldrich, St. Louis, MO), 0.8g NaH_2_PO_4_ (Sigma Aldrich), and 8.5g NaCl (Fisher Scientific) into 1L of DI water, pH was adjusted to 7.6, then the solution was autoclaved. Blue phage buffer (BPB) was made by dissolving 6.057g Tris (ThermoFisher Scientific, Waltham, MA), 20.33g MgCl_2_ • 6H_2_O (ThermoFisher Scientific), and 1mL of a 1% w/v Coomassie brilliant blue G-250 solution (Alfa Aesar) into 1L DI water, pH adjusted to 7.5, then autoclaved.

### Phage propagation and plaque assays

For plaque and lysogen assays, the *E*. *coli* O157:H7 C7927 isolate from contaminated apple cider was used as the host organism for the various ɸV10 strains [25]. Wildtype ɸV10 phage were propagated from the natural lysogen *E*. *coli* Strain 10 [26] and ɸV10*nLuc* were propagated from the *E*. *coli* O157:H7 C7927 lysogen for the phage [16].

Plaque assays were performed using the soft agar overlay method [27]. ɸV10 plaque assays used LB agar plates and molten, sterile LB top agar for overlaying, while ɸV10*nLuc* plaque assays used LB agar plates and molten, sterile modified LB top agar for overlaying in order to better visualize plaques. The following day, for plates containing between 20 and 500 plaques: plaques were enumerated and titers were determined by multiplying the plaque count by the dilution factor and averaging the values of replicates.

### Printing and dissolvable papers

A commercially available sodium carboxyl methyl cellulose dissolving paper, SmartSolve IT117138 (SmartSolve Industries, Bowling Green, Ohio) was used as the medium for deposition of ɸV10. Printing of phage was done using a piezoelectric inkjet deposition platform, PipeJet (BioFluidiX, Freiburg, Germany). To precisely control the printed volume of bio-ink on the substrate, printing parameters (stroke and stroke velocity) were tuned to obtain consistent droplet volume without satellites during the ejection process. An image analysis method [28] was used to evaluate the average diameter of droplets after impacting the substrate. Phage were deposited onto 1cmx1cm pieces of dissolvable paper with the following printing parameters: print frequency = 10Hz, stroke = 100%, stroke velocity = 90μm*ms^-1^, droplet volume = 20nL, media advance speed = 2 mm per s, media return speed = 20mm per s, nozzle diameter = 200 μm, standoff distance between print head and substrate = 1mm.

### Storage and evaluation of phage-functionalized paper

Initial evaluation of applying phage to dissolvable papers was carried out by: deposition of 1.75μL of phage onto dissolvable paper, storage of papers at 23°C for two hours, then placement of papers in sterile Petri dishes, sealing with Parafilm, and placement at 4°C overnight. Surface area of phage distribution patterns on papers was determined by measuring diameters of the circular droplets via digital calipers (Speedway Motors, Inc., Lincoln, NE). To compare surface area differences between the five-droplet and one-droplet patterns, a two-tailed T-test (with equal variance not assumed) was performed. For evaluation of remaining viable phage, phage papers were added to 1mL sterile, deionized water in sterile Eppendorf tubes and vortexed for 10 seconds until paper had completely dissociated. Homogenized samples were then diluted serially in BPB and used in plaque assays.

Evaluation of effects of varied drying time of phage on dissolvable papers was carried out by depositing 1.75μL of phage onto 1cm x 1cm dissolvable paper, storage at 23°C for the allotted time in a sterile Petri dish, then immediate dissolution of the phage paper in 1mL sterile, deionized water with vortexing. Once papers were completely homogenized in water, serial dilutions in BPB and plaque assays were performed. The log-reduction for each treatment was calculated by converting the average titer of a treatment group into log_10_ and subtracting this number from the average titer of the control group (1.75μL of phage into 1mL sterile, deionized water without dissolvable paper), also converted to log_10_.

### Longevity study with dissolvable paper and ɸV10

Two liquid stocks of ɸV10 with similar titers were used: one consisting of phage stored in BPB and the other stored in BPB + 0.5M sucrose. Fifteen 1cm^2^ pieces of dissolvable paper were used for each treatment group onto which 1.75 μL of phage stock was pipetted to the center of each paper. Each phage paper was allowed to dry in a sterile petri dish at 23°C for five minutes, then three of each paper was placed in a new petri dish, sealed with Parafilm, and stored at 4°C until the day of testing for a group of papers. Each day of testing, one petri dish with three phage papers for each treatment was removed from refrigeration and each paper was dissolved in 1mL sterile, DI water. Controls consisted of 1.75 μL of respective phage stocks being added to 1mL sterile, DI water and subsequent dilutions.

### Lyophilization of phage

For lyophilization experiments, 100 μL samples of phage were added to 2mL cryovials (Thomas Scientific, Swedesboro, NJ). The caps for the tubes were kept off and the tops of the tubes were covered with Parafilm. Five holes were pierced in each piece of parafilm with a 23Ga needle to allow for evaporation. Samples were frozen at -80°C overnight and then immediately transferred to a freeze-drying jar and attached to a Labconco Freezone 6 freeze drying unit (Labconco Corporation, Kansas City, MO). Freeze drying was carried out for 48 hours.

### Longevity study with lyophilized ɸV10

Phage stocks of ɸV10 were prepared using BPB and BPB plus two different concentrations of sucrose: 0.1M and 0.5M. Aliquots of 100 μL of each phage stock were pipetted into cryotubes (30 tubes per treatment). Screw-top caps were left off the tubes and they were instead covered with Parafilm (Bemis Company, Inc., Wisconsin) with small holes pierced into the film. Tubes were then frozen at -80°C for 16 hours and lyophilized. After lyophilization, caps were replaced upon the tubes and all tubes were stored at room temperature in a desiccator. The original liquid stocks of phage were maintained at room temperature as well and were used as controls for viable phage numbers at the various time points.

For each time point, three tubes of freeze-dried phage for each treatment group (BPB, BPB+0.1M sucrose, and BPB+0.5M sucrose) were removed from the desiccator and reconstituted in 1mL sterile, DI water at 23°C. Control samples were generated in triplicate by aliquoting 100 μL of the liquid phage stock (with or without additives) into 900 μL sterile, DI water at 23°C. All samples were vortexed and serially diluted in BPB and subjected to plaque assays for enumeration. At each time point, three new tubes of each treatment group were evaluated for phage viability; this increases potential for sample-to-sample variation between time points, but most appropriately evaluates the effect of freeze drying.

### Lysogen assays with ɸV10*nLuc*

An overnight culture of *E*. *coli* O157:H7 C7927 was diluted to achieve a multiplicity of infection (MOI) of approximately 10:1 based on the titer of the phage stock being used. 100 μL of ɸV10*nLuc* solution, 100 μL of *E*. *coli* O157:H7 C7927 dilution, and 800 μL of LB broth were aliquoted into a sterile Eppendorf tube. The tube was vortexed briefly and incubated at 37°C for 40 minutes. Serial dilutions from the tube were performed after incubation and 100 μL of each dilution was spread plated onto LB + Kanamycin agar. The following day, Kan^R^ colonies were enumerated. The rate of lysogeny was calculated by dividing the number of Kan^R^ colonies by the titer of the phage stock used to generate the lysogens.

### Statistical analysis

All titer and rate of lysogeny data were generated by experiments performed in triplicate. Data were analyzed using a one-way ANOVA analysis. When evaluating lyophilization longevity study data, one-way ANOVA analysis was conducted with the homogeneity of variance not assumed, followed by a Games-Howell post-hoc analysis [29]. Analyses were carried out using SPSS statistical software package (IBM Corporation, Armonk, NY). All statistical conclusions were based on the p value cutoff of 0.05.

## Results and discussion

### Printing of phage onto dissolvable papers

**Fig 1:**
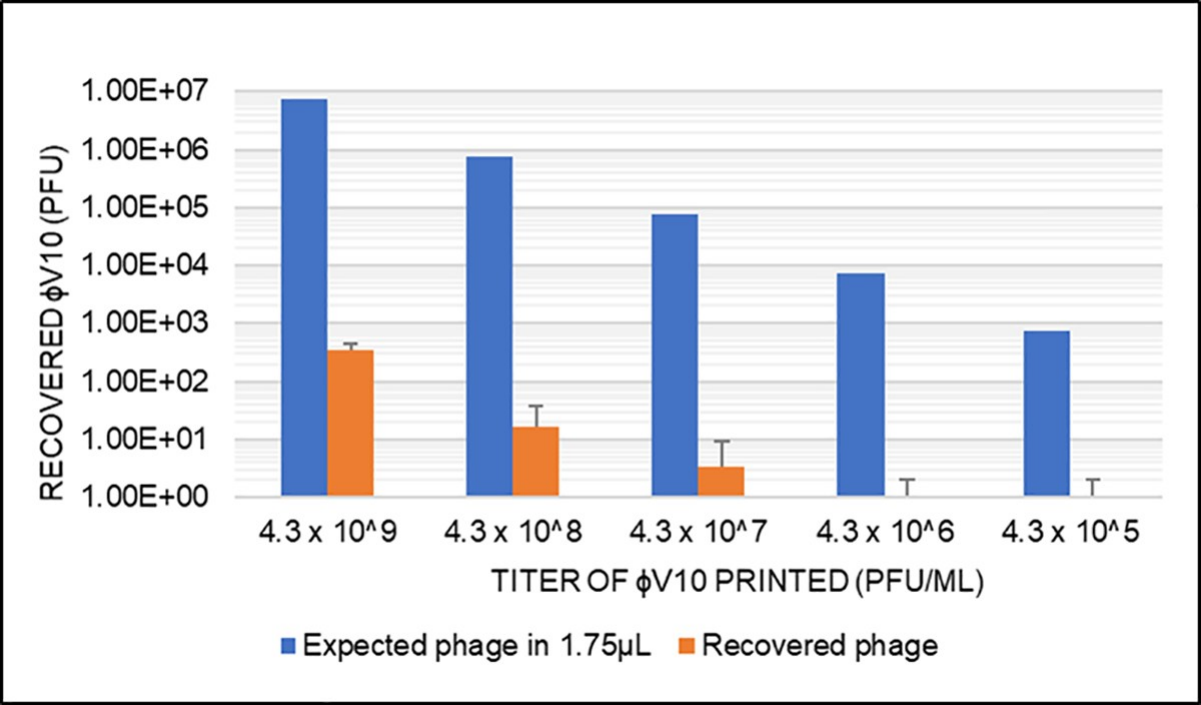
Viable ɸV10 recovered from printing onto dissolvable paper. Five stock concentrations of ɸV10 were printed onto 1cm^2^ pieces of dissolvable paper. Upon dissolution and dilution, the illustrated number of viable ɸV10 were recovered. The left column for each treatment group represents a theoretical 100% recovery, whereas the right column describes the number of viable phage experimentally recovered.

Initial work was done to evaluate the maximum amount of phage that could be deposited onto and recovered from a 1cm^2^ piece of dissolvable paper using a nanoliter printing device without compromising the integrity of the paper. Various concentrations (4.3 x 10^9^, 4.3 x 10^8^, 4.3 x 10^7^, 4.3 x 10^6^, and 4.3 x 10^5^ pfu*mL^-1^) of a stock of phage in blue phage buffer (BPB) were used to determine if an irreversible interaction occurred between the paper and the phage. Results in **Fig 1** show substantial loss of viable phage for each of the dilutions as the resulting plaque numbers represent a more than four-log loss of viable phage as compared to a liquid stock control. Resulting loss of recoverable, viable phage could be a result of inactivation of phage, a physical interaction between phage and fibers of the paper, and/or from sheer forces from the printing nozzle.

As sheer forces through a printing nozzle may affect biological entities in solution [30], a simple experiment was undertaken to evaluate loss of viable phage after printing. Results from this experiment showed no loss in viable phage after ejecting 500 μL of a liquid stock of phage through the printing nozzle and immediately performing serial dilutions and plaque assays (data not shown). These data taken together, led to the hypothesis that the 2-dimensional density with which the phage were applied to the dissolvable paper directly affected the number of viable phage that were able to be recovered. The method by which phage became sequestered or inactivated was beyond the scope of this work but would make for a valuable follow-up study to determine the mechanism by which phage are unable to be recovered from this physical medium.

**Fig 2:**
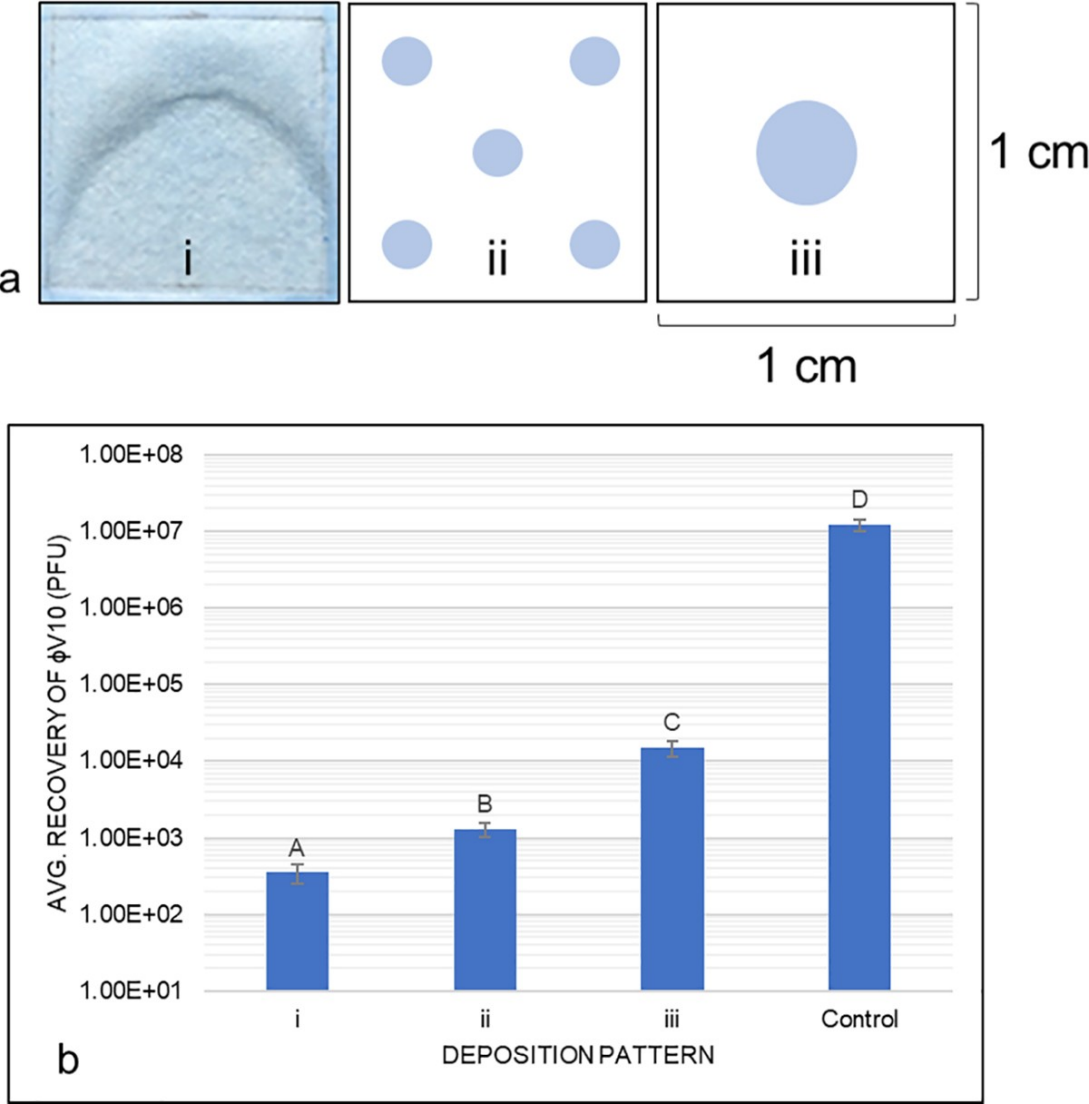
Recovery of ɸV10 applied to dissolvable paper in different deposition patterns. (A) Deposition patterns of ɸV10 onto 1cm^2^ dissolvable paper. A consistent volume (1.75 μL) of liquid phage stock applied to papers in three patterns. i) ɸV10 printed onto dissolvable paper across the entire surface of the paper using 10nL droplets in a grid-like pattern of deposition (surface area of 100 mm^2^). ii) Five droplets containing 350 nL were pipetted onto the paper in approximately equal spacing (surface area average of 21.24 ± 2.94 mm^2^, n = 15). iii) One droplet containing 1.75 μL was pipetted in the center of the dissolvable paper (surface area average of 17.78 ± 2.23 mm^2^, n = 15). (B) Recovery of ɸV10 applied to dissolvable paper in different deposition patterns. Deposition patterns correspond to the 1.75 μL patterns listed in Fig 2A. Significant differences between recovered PhiV10 from various deposition patterns onto dissolvable papers are a result of a one-way ANOVA analysis with post-hoc Duncan test. Capital letters denote significantly distinct (p < 0.05) titer values as a result. Distinct differences in recovered viable phage counts are seen and are inversely proportional to the surface area of phage applied to papers. The control group were 1.75 μL of the same phage stock into 1mL sterile water. Results shown are from three replicates of each treatment.

In an effort to improve efficiency of recovering viable phage from dissolvable paper, the effect of density of deposition of phage was evaluated. Distribution of 1.75 μL of phage onto 1cm^2^ dissolvable papers in different patterns (**Fig 2A**) showed an increase in recoverable, viable phage with decreasing surface area (**Fig 2B**). Of the three distribution patterns, one central droplet of phage solution generated the greatest number of viable phage upon dissolution of the paper into 1mL of water. The surface areas of the one central droplet and five dispersed droplet patterns, while numerically similar, were statistically different (two-tailed t-test with unequal variance assumed, p-value = 0.0006, alpha = 0.05). The viable phage recovery and associated surface areas suggest that either phage are irreversibly binding to fibers of the paper (and thus fewer are available to infect host cells in plaque assays when spread out across larger areas of paper) or that more thorough drying of the phage is detrimental to their viability (as a larger surface area on the paper allows for more air contact and thus, drying). Jonczyk et al. (2011) have previously described that drying or desiccation of phage may severely limit their viability, which prompted investigation into the effect drying has on ɸV10 [31].

Until this point in this research, phage were applied to dissolvable papers and allowed to dry for two hours at 23°C and subsequently refrigerated overnight at 4°C until tested the following morning. In order to evaluate the effect drying had on phage deposited onto dissolvable paper, storage parameters were varied, and dissolvable papers were then evaluated for surviving viable phage. The data in **Table 1** describe the improved recovery of viable phage with less drying time after applying phage to the dissolvable papers. After only ten minutes of drying at 23°C, a two-log reduction in titer was observed with greater drying times resulting in greater loss of viable, recoverable phage. By comparing titer reductions between time points, the sensitivity of ɸV10 to desiccation becomes apparent. This phenomenon has been shown before in other phage using several storage conditions, however, it can potentially be mitigated by adding stabilizing additives to the liquid phage stock before drying [32]. Thus, sucrose, an inexpensive and commonly used stabilizer for protein and phage stabilization during drying was chosen to attempt to stabilize ɸV10 when applied to dissolvable paper [33, 34].

**Table 1:** Viable ɸV10 phage recovered from dissolvable papers after varying time of drying.

Time dried (min)	Avg. titer (log pfu*mL^-1^)	Log_10_ Reductions
Control	7.474 ± 0.02 *a*	--
5	6.668 ± 0.11 *b*	0.806
10	5.290 ± 0.29 *c*	2.184
20	4.917 ± 0.03 *d*	2.557
30	5.016 ± 0.11 *cd*	2.458
45	4.698 ± 0.18 *de*	2.776
60	4.493 ± 0.42 *e*	2.981
90	4.503 ± 0.09 *e*	2.971
120	4.424 ± 0.15 *e*	3.050

Resulting titers of dried phage papers are described. Average titer volumes are mean ± standard deviation, n = 3. Different italic letters indicate a significant difference in this column as a result of one-way ANOVA analysis and post-hoc Duncan test (p < 0.05). Each letter represents a significantly different grouping when comparing the titers resulting from each of the treatment conditions.

**Fig 3:**
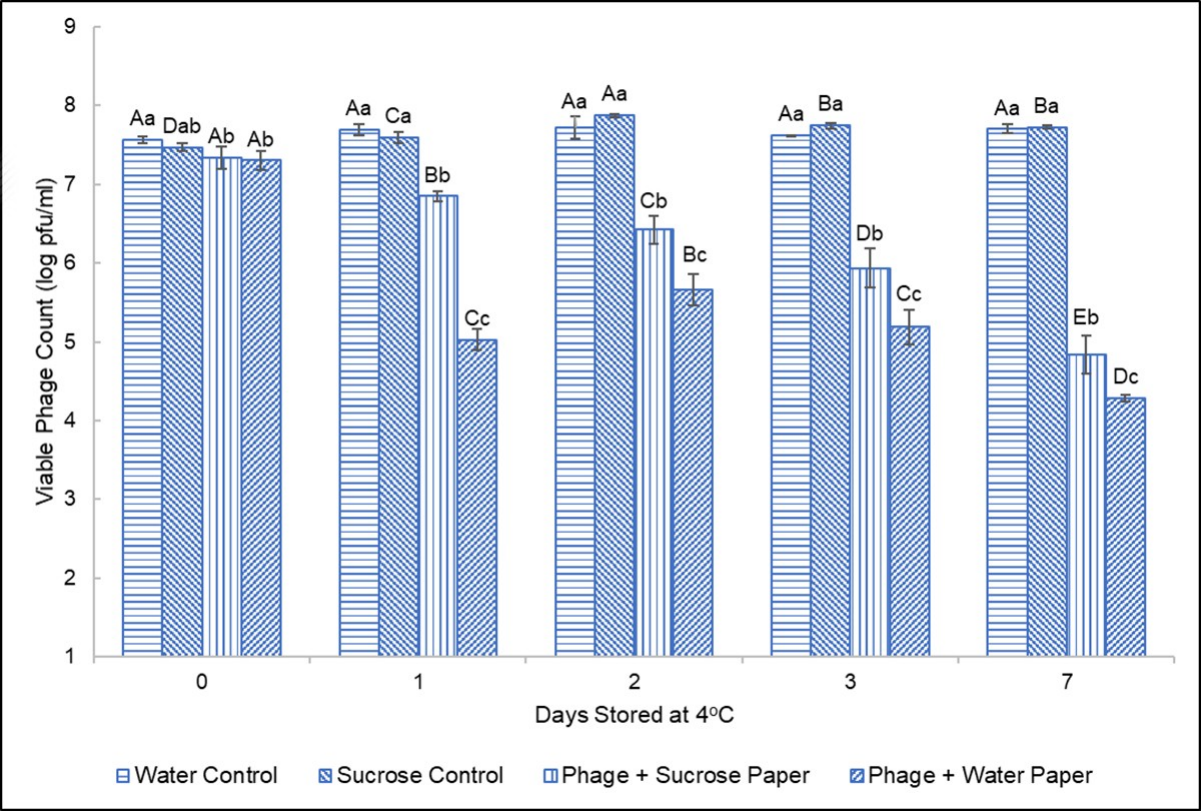
Longevity of ɸV10 on dissolvable papers stored at 4°C. Data shown include one-way ANOVA analysis to show significant (p < 0.05) differences between treatment groups at each timepoint and within each treatment group over time. Lowercase letters denote significant differences between treatments within a single timepoint. For each timepoint, the lowercase letter “a” corresponds to the highest, statistically distinct value for recovered phage titer, with “b” representing the second highest, statistically distinct value, etc. Capital letters denote significant differences within a single treatment group over time. Capital letters are only significant within a single treatment group over time and are assigned in order of descending titers (e.g. “A” is always the largest, significantly different titer for a single treatment group).

A shelf-life study was performed to evaluate the stabilizing effect sucrose had on phage being applied to dissolvable papers. Over the course of seven days, the viable phage count was assessed for each treatment group (**Fig 3**). Upon initial testing on day 0, a slight, yet statistically significant, difference was observed between the liquid stock controls and the phage-impregnated papers. After one day of storage at 4°C, significantly fewer viable phage were recovered from phage immobilized on dissolvable paper compared to liquid stock controls. However, between the phage with and without sucrose applied to the paper, significantly more viable phage were able to be recovered from phage with 0.5M sucrose at each remaining timepoint. After seven days, the BPB and BPB+0.5M sucrose papers showed 3.02-log and 2.50-log reduction in viable phage when compared to their liquid stock controls, respectively. This reduction represents significant loss over time of phage papers stored at 4°C, with the addition of sucrose to the phage solution contributing a significant benefit to phage viability compared to the absence. After optimizing distribution patterns, storage conditions, and osmoprotectant presence, it was concluded that this approach to packaging ɸV10 was not suitable for potential commercialization. The poor phage recovery rate after just seven days of refrigerated storage prompted investigation into an alternative approach to functionalizing this particular phage.

### Lyophilized phage shelf-life study

**Fig 4:**
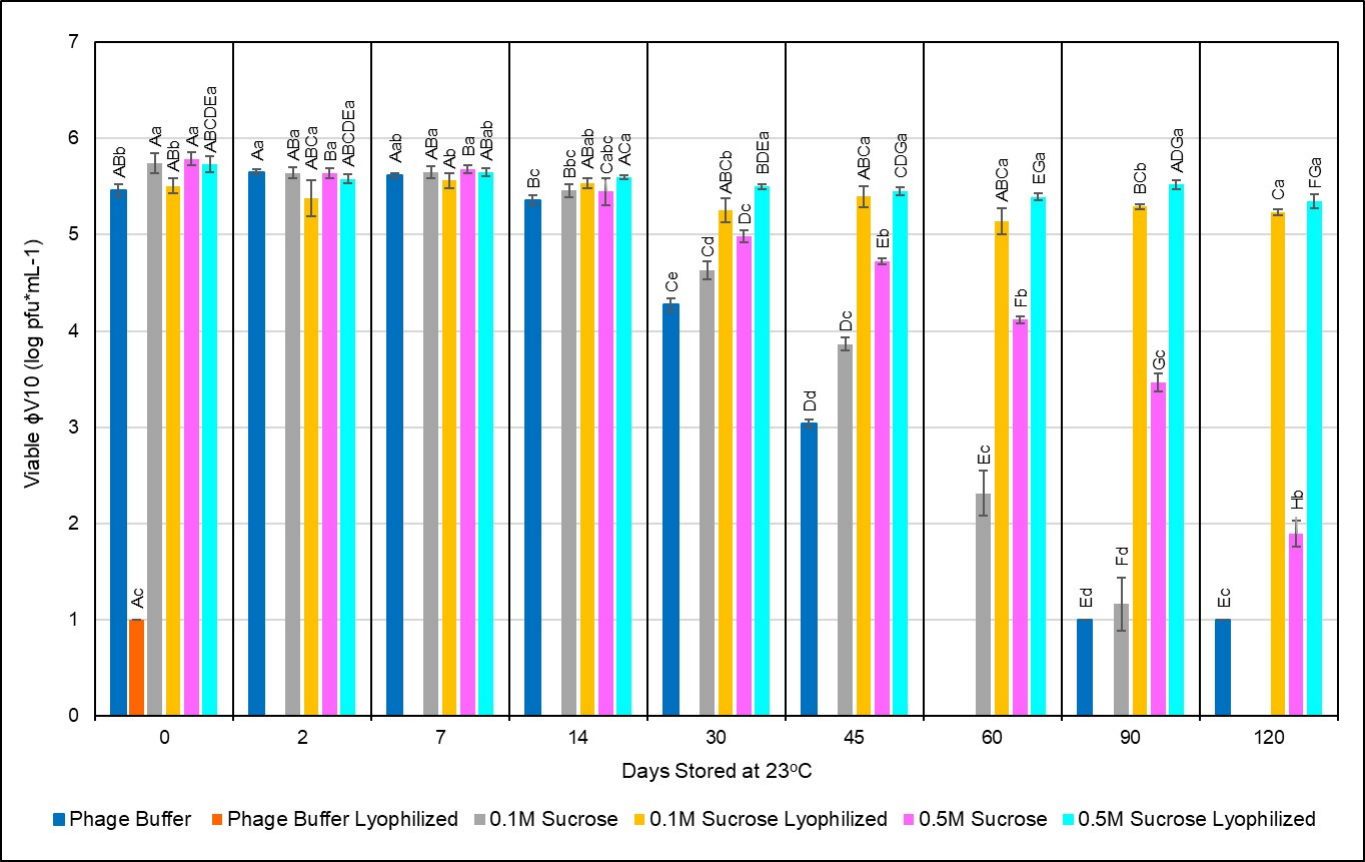
Longevity of lyophilized ɸV10. Titers from treatment groups of ɸV10 stored in BPB with varying concentrations of sucrose with or without lyophilization. One-way ANOVA analysis has been applied to illustrate significant differences between viable phage titers between treatment groups over time. Each lowercase data label corresponds to a significantly different (p < 0.05) titer value within each timepoint. These lowercase labels signify statistically distinct titer values between the different treatments only within a single timepoint. Capital letters denote significantly distinct (p < 0.05) titer values for a single treatment and time point compared to each other time point within the same treatment conditions. These capital data labels correspond to how recovery of phage from a single treatment changed over time; each capital letter denotes a statistically distinct titer value for a single treatment over several timepoints.

As previous experiments in this study have shown an improved resistance to drying ɸV10 in the presence of sucrose, sucrose was also evaluated for its ability to protect viable ɸV10 from the harsh conditions of lyophilization. This experiment focused on the evaluation of phage viability over time while lyophilized samples and liquid stock controls were stored at room temperature (23°C). The data from this 120-day analysis (**Fig 4**) revealed several interesting findings. First, the samples lyophilized in only BPB were all but entirely inactivated by the lyophilization process as immediately upon reconstitution, a five-log reduction in titer was observed. This outcome was foreshadowed by the sensitivity of ɸV10 to desiccation in the dissolvable paper experiments and illustrates the need to study individual phage responses to processing methods. Through the first seven days of incubation at 23°C, all three of the liquid stocks of phage and the 0.1M and 0.5M sucrose lyophilized samples maintained similar levels of viability. After 30 days of storage at 23°C, however, significant differences were observed between each of the liquid stocks of ɸV10 and the two sucrose-containing lyophilized samples as indicated by the lowercase data labels in Fig 4. The significant differences at this point begin to indicate the relative stability of each formulation of phage at ambient storage temperatures. As storage time increased, differences between liquid stocks of phage and lyophilized samples with sucrose also increased, culminating a three-log difference in titers after 120 days. By comparing significant differences in recoverable ɸV10 between treatments at a given time (lowercase significance indicators in Fig 4), it becomes clear that lyophilization with sucrose confers recovery advantages over their liquid buffered control counterparts. Additionally, the presence of 0.1M or 0.5M sucrose in lyophilized ɸV10 samples generated negligible differences in recoverable phage, indicating the sufficiency of using the lower concentration of cryoprotectant for effective processing.

When comparing viable titers within the same treatment group over time (capital letter data labels in Fig 4), clear trends are evident for each treatment. Titers for all surviving treatments showed no statistical difference when compared to their respective controls after seven days of storage at 23°C. After 14 days, the 0.1M sucrose + BPB and 0.5M sucrose + BPB liquid samples showed their first significant decline in titer. However, after 30 days, all the liquid stocks showed significant declines in titer while the two lyophilized sucrose samples showed no significant differences in their titers compared to their respective day 0 controls (p < 0.05). The final timepoint of 120 days was the first instance in which the 0.1M sucrose and 0.5M sucrose lyophilized samples showed a significant reduction in titer: losing 0.27- and 0.38-log pfu*mL^-1^, respectively, over the entire 120-day period. Together, these data show that lyophilizing ɸV10 with 0.1M and 0.5M sucrose is a more effective method than traditional phage storage buffer for preserving the phage at room temperature for at least 120 days. These findings suggest that integrating temperate phage, such as ɸV10, into commercial pathogen detection kits may have success with processing lyophilized phage using sucrose as a stabilizing agent. As has been suggested in literature, finding and evaluating methods for long-term, anhydrous storage of phage will be critical in translating their laboratory-based capabilities into successful societal tools [32].

### Lyophilization of ɸV10*nLuc*

A similar lyophilization experiment was performed with the recombinant reporter phage ɸV10*nLuc* after positive results from the wildtype ɸV10 experiments. The effects of lyophilization on the ability for ɸV10 to produce lysogenic infections are possible with a recombinant reporter phage due to the resulting selectable phenotype of lysogens. Maintaining the high rate of lysogeny of this reporter phage is essential for any technique that would be included in its commercial production. As with the lyophilization experiment with the wildtype strain, the number of viable phage was initially determined for each treatment group by performing plaque assays.

**Fig 5:**
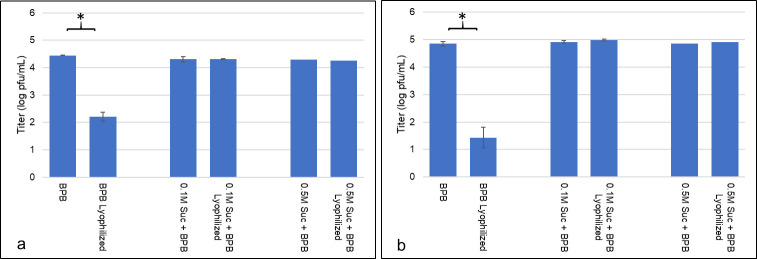
ɸV10*nLuc* titers after lyophilization and storage at 23°C. (A) Initial ɸV10*nLuc* titers after lyophilization. For each lyophilized treatment, titers were compared to their respective liquid buffer controls. Asterisks denote statistical significance between groups (p < 0.05). (B) ɸV10*nLuc* titers after lyophilization and 18 days of storage at 23°C. For each lyophilized treatment, titers were compared to their respective liquid buffer controls. Asterisks denote statistical significance between groups (p < 0.05).

**Fig 6:**
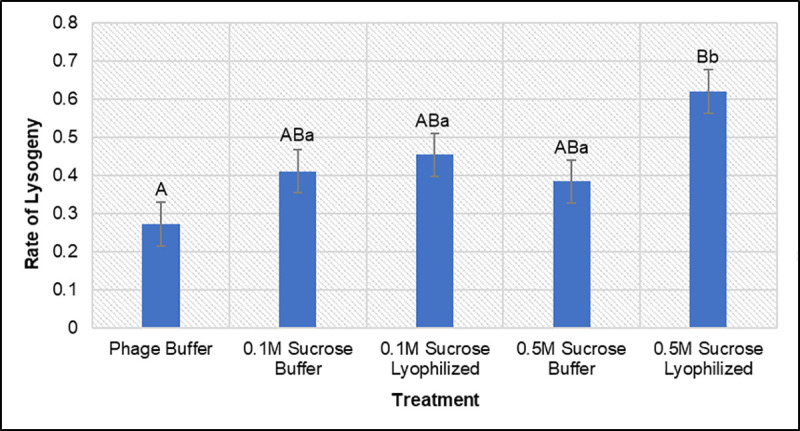
Rates of lysogeny for ɸV10*nLuc* in liquid and lyophilized buffers after 18 days. The rates of lysogeny are determined by averaging triplicate experiments where the number of kanamycin resistant lysogens that are formed for a given phage sample are divided by the total number of phage in the sample determined by plaque assays. Capital letters denote a sample’s statistical grouping compared to the phage buffer control group. Lowercase letters denote significant differences between all treatment groups (e.g. 0.1M sucrose buffer and lyophilized 0.1M sucrose buffer samples).

Data from day zero and day 18 testing showed similar results to the wildtype experiment (**Fig 5**), which was expected due to the only differences between the two phage being recombination of the reporter gene construct into a non-structural gene [16]. Lyophilized phage stored in only BPB showed a significant decrease in the number of viable phage, while lyophilized samples containing BPB + sucrose were not significantly affected by the lyophilization process. After 18 days, these same trends remained evident as only slight, insignificant differences were observed between the lyophilized samples with sucrose and their respective controls. Lyophilized ɸV10*nLuc* samples showed similar responses in the number of viable phage to their wildtype counterparts, illustrated by plaque assay data. Lysogen assays from the surviving treatment groups of ɸV10*nLuc* (**Fig 6**) revealed that none of the lyophilized samples with sucrose performed significantly worse than the phage stored in liquid phage buffer after 18 days. In fact, the lyophilized phage with 0.5M sucrose showed a statistically higher rate of lysogeny than the liquid BPB control. These findings indicate that lyophilization is an acceptable method for processing ɸV10, and its many reporter phage configurations, in a shelf-stable manner while preserving relative titer and rates of lysogeny of the phage.

In summary, the need for shelf-stable processing methods for bacteriophage is increasing as bacteriophage become more integrated into the food safety and human health sectors. The data here describe the limitations and key criteria to consider with respect to applying temperate bacteriophage to dissolvable paper. Additionally, lyophilization of ɸV10 in the presence of sucrose appears to be a viable method for future packaging considerations in commercial products centered on temperate phage. As more temperate phage-based bacterial detection platforms are brought to market, stable storage requirements will prove critical to commercial success.
